# Degradation kinetics of postharvest quality attributes of rocket leaves grown under different nitrogen rates

**DOI:** 10.1002/jsfa.70960

**Published:** 2026-08-09

**Authors:** Aysha Saleem, Maria Luisa Amodio, Pramod V Mahajan, Maria Gonnella, Lucia Bonelli, Giancarlo Colelli

**Affiliations:** ^1^ Dipartimento di Scienze Agrarie, Alimenti, Risorse Naturali e Ingegneria Università di Foggia Foggia Italy; ^2^ Department of Systems Process Engineering Leibniz‐Institut für Agrartechnik und Bioökonomie e.V. (ATB) Potsdam Germany; ^3^ Institute of Sciences of Food Production, CNR‐National Research Council of Italy Bari Italy

**Keywords:** rocket leaves, nitrogen fertilization, postharvest quality, kinetics of degradation, shelf‐life modelling

## Abstract

**BACKGROUND:**

*Diplotaxis tenuifolia* L. cv Dallas (rocket leaves) is one of the most widely consumed fresh‐cut salad vegetables in Europe, especially in southern Italy, being appreciated for its pungent taste and health promoting phytochemicals. However, its short postharvest shelf‐life is still a major drawback. In this study rocket plants were grown in pots using a soilless peat/perlite substrate (3:1, *v/v*) under glasshouse conditions and irrigated with nutrient solutions containing six nitrogen (NO_3_^−^ and NH_4_^+^) concentrations (0.005–0.015 mol L^−1^) and the quality characteristics and shelf‐life of rocket leaves were evaluated. Samples were kept at 5 °C in passive modified atmosphere packaging with clamshells in 99% relative humidity for 18 days. Quality parameters such as vitamin C, chlorophyll, firmness, microbial load (mesophilic aerobic bacteria, yeasts and moulds) and general sensory acceptability parameters were monitored at regular intervals. Degradation kinetics were explained by primary (first‐order and lag‐exponential) and secondary models and predicted sensory shelf‐life at a threshold score value 3 for marketability.

**RESULTS:**

Results showed that low to moderate nitrogen levels had significantly higher vitamin C content and sensory quality. In contrast, greater nitrogen levels (> 0.013 mol L^−1^) promoted nutrient loss at faster rates and surpassed the European safety levels for nitrates. Shelf‐life prediction models showed that they fit well (*R* ^2^ > 0.90) and indicated a trade‐off between quality preservation and nitrate safety.

**CONCLUSION:**

Moderate nitrogen fertilization (corresponding to 0.005–0.007 mol L^−1^) favoured both the quality retention and the compliance with the regulations, in favour of sustainable agricultural practices. © 2026 The Author(s). *Journal of the Science of Food and Agriculture* published by John Wiley & Sons Ltd on behalf of Society of Chemical Industry.

## INTRODUCTION

*Diplotaxis tenuifolia* (rocket) is a widely used leafy vegetable in the Mediterranean regions, which is eaten fresh and cooked and is valued due to its intense pungent taste and good amounts of health‐promoting substances such as vitamin C, carotenoids, glucosinolates, phenolics, and minerals.^1^ In 2020 southern Italy had an area of about 3500 ha of rocket growing in it and an annual product of 400 000 tons.^2^ Minimal processing salads are in increasing demand by consumers who buy ready‐to‐eat salads that contain low amounts of processing; the rocket leaves alone are taking approximately 10% of this market.^3^

Nevertheless, rocket is not an exception, and it belongs to the category of vegetables regulated by a limit of nitrate content as stated by the European Commission Regulations No. (EU) 2023/915.^4^ The strategies of nitrogen fertilization, light intensity, temperature, genotype and the initial level of soil/water nitrate are the factors that greatly affect nitrate accumulation. Having the nitrate concentration exceeding the limits does not only harbor the possible health hazards to the consumers but also puts the producers at risk of lost export. Incidentally, the lack of nitrogen may decrease chlorophyll content, deteriorate the leaf colour, and affect the general acceptance by the consumers in the storage stage.^5^

Depending on the preharvest conditions, handling, processing, and storing temperature, fresh‐cut rocket leaves have an average shelf‐life of 7–14 days.^6^ The primary limiting agents are chlorophyll degradation that caused yellowing, tissue softness, and development of off‐odour that together causes the loss of the visual attractiveness and the appeal as a whole.^7^ According to recent research, preharvest fermentation activities and postharvest management are key determinants of the final products in terms of quality and shelf life.^8^ However, the majority of the predictive shelf‐life models put emphasis on the postharvest factors, but there is little incorporation of agronomic factors, including nitrogen fertilization. Recent research has pointed out the necessity of mechanistic models that can be used to access preharvest inputs and postharvest quality, in particular with regard to nitrate‐sensitive leafy vegetables.^9^, ^10^, ^11^, ^12^ Such models have the potential to inform both the growers and the supply chain operators in the process of balancing the yield, safety, and sustainability. Specifically, the interactive effects of preharvest nitrogen levels on postharvest degradation kinetics of various quality attributes, including the critical safety parameter of nitrate content, need to be systematically studied and modelled.

Therefore, the objective of this study was to evaluate the effect of six distinct nitrogen fertilization levels on the postharvest quality and shelf‐life of rocket leaves stored under passive modified atmosphere conditions. Further utilize our findings to build kinetic models to predict the shelf‐life based on degradation rates. This integrative approach aims to validate predictive tools for defining fertilization strategies that optimize quality attributes while ensuring regulatory compliance.

## MATERIALS AND METHODS

### Raw materials and experimental design for the kinetic analysis of quality degradation

Rocket plants (*Diplotaxis tenuifolia* L. cv Dallas, ISI Sementi, Fidenza, PR, Italy) were grown in an unheated polymethacrylate glasshouse at six different nitrogen fertilizer levels (T_1_ = 0.005, T_2_ = 0.007, T_3_ = 0.009, T_4_ = 0.011, T_5_ = 0.013, T_6_ = 0.015 mol L^−1^; resulting by the combination of ammonium and nitrates, as reported in Table 1) at the experimental farm ‘La Noria’ of the Institute of Sciences of Food Production – National Research Council (Mola di Bari, Italy, 41°03′ N, 17°04′ E; 24 m above sea level).

**Table 1:** Fresh weight, leaf area and nitrate content in rocket leaves grown in soilless cultivation at increasing nitrogen rates

Treatment	Supplied nitrogen		Nitrogen fertilizer		% of nitrate (NO_3_^−^)	Fresh weight	Leaf area	Nitrate first harvest	Nitrate last harvest
	(mol L^−1^)	(mg L^−1^)	N‐NO_3_ (mg L^−1^)	N‐NH_4_ (mg L^−1^)		(g per pot)	(cm^2^ per pot)	(g kg^−1^ FW)	(g kg^−1^ FW)
T_1_	0.005	70	66	4	94	228 ± 7.17^c^	1318 ± 20.74^c^	3.50 ± 0.39^b^	3.62 ± 0.77^c^
T_2_	0.007	98	80	18	82	298 ± 16.18^bc^	1634 ± 101.72^bc^	6.55 ± 0.77^a^	5.21 ± 0.67^bc^
T_3_	0.009	126	94	32	75	359 ± 19.42^ab^	1960 ± 111.95^ab^	5.87 ± 0.02^a^	8.29 ± 0.95^ab^
T_4_	0.011	154	108	46	70	407 ± 29.99^a^	2148 ± 92.14^a^	5.86 ± 0.41^a^	8.82 ± 0.85^ab^
T_5_	0.013	182	122	60	67	433 ± 43.26^a^	2238 ± 127.86^a^	6.11 ± 0.52^a^	11.07 ± 0.85^a^
T_6_	0.015	210	136	74	65	450 ± 4.85^a^	2370 ± 43.58^a^	6.87 ± 0.37^a^	12.14 ± 0.91^a^

*Note*: Data relating to fresh weight (FW) and leaf area are expressed in reference to the production of a 4.5 L‐pot (20 cm in diameter) in which the rocket plants were grown. Different letters within the same column indicate significant differences between treatments.

Seedlings were transplanted on 22 December 2021 into plastic pots (4.5 L volume) containing a peat/perlite mixture (3:1, *v/v*) as substrate. Plants received nutrient solutions (NSs) containing nitrogen at the specified levels (Table 1). The complete NS composition included phosphorus (1.1 mmol L^−1^), potassium (4.3 mmol L^−1^), magnesium (1.7 mmol L^−1^), calcium (2.9 mmol L^−1^), sulphur (3.4 mmol L^−1^), iron (0.023 mmol L^−1^), copper (0.001 mmol L^−1^), manganese (0.004 mmol L^−1^), zinc (0.002 mmol L^−1^), boron (0.025 mmol L^−1^), and molybdenum (0.0005 mmol L^−1^).

The experimental design was a randomized block design with three biological replications. The NS was applied via drip irrigation emitters (one emitter per pot), set to deliver solution for 1 min, four times per day. Pots were arranged on trough‐benches, with each elementary experimental unit (block) containing 20 pots. The NS was managed in an open cycle system, meaning no recirculation of the NS occurred.

Multiple harvests were conducted to ensure a comprehensive evaluation across different growth periods: starting from 2 February 2022, and with subsequent cuttings in the following months (March, April for two separate harvests, and May). This resulted in five distinct harvest events from the same planted individual. Freshly harvested rocket leaves from each treatment and replicates were immediately transported within 2 h to the postharvest laboratory at the University of Foggia, Italy. Upon arrival, leaves were briefly air‐dried at room temperature to remove excess surface water if any and stored as it was with medium stems attached and leaf blades intact.

Twenty‐seven clamshell containers (polypropylene, perforated, 250 mL volume capacity) each containing 100 g of leaves were prepared for each of the six nitrogen treatments, resulting in 162 clamshells in total. Three clamshells per treatment were immediately used for initial (day 1) assessment of quality parameters and remaining were stored under controlled condition with humidify flow of air at 5 °C (99 % relative humidity (RH)) for 18 days, to avoid any affect due to the modification of the gas atmosphere. On each of the eight sampling days during storage (days 1, 3, 4, 7, 10, 14, 16, and 18), three clamshells per treatment were opened for destructive sampling. The variability reported in this study is the variation among these three biological replicates. Primary and secondary kinetic models were fitted using the mean values obtained across the three biological replicates and five harvests (*n* = 15) total biological replicates per data point for each of the six nitrogen fertilizer treatments. This approach was chosen to prevent environmental exposure, cross‐contamination (particularly for microbial analysis) and to avoid physical damage or altered respiration from repeatedly opening and handling the same package. The sampling order was kept consistent, starting with microbiological analysis, followed by firmness, colour, and chemical analyses (such as vitamin C). For every parameter evaluated, three replicates were taken from the opened clamshell (one from each three) to determine the mean values and associated experimental variability.

All harvests followed identical postharvest handling and storage procedures, allowing the kinetic modelling to incorporate the natural physiological variability occurring across the production season. Cold storage was conducted in complete darkness to prevent light‐induced physiological changes and ensure consistent postharvest conditions across all harvests.

### Nitrate determination

Nitrate concentration was measured at the initial harvest (first harvest, 2 February 2022) and the final harvest (last harvest, May 2022) to evaluate its accumulation over the growing season and in response to nitrogen fertilization. It was determined by ion exchange chromatography technique (IC‐Dionex DX120; Dionex Corporation, Sunnyvale, CA, USA) with a conductivity detector, according to D’Imperio *et al*.^13^ Briefly, freeze‐dried leaf material was treated with solution of sodium carbonate (Na_2_CO_3_, 3.5 mmol L^−1^) and sodium hydrogen carbonate (NaHCO_3_, 1 mmol L^−1^), for 30 min, at room temperature. The extracts were diluted and filtered by using 0.45 μm (RC filter) followed by a Dionex OnGuard IIP (Thermo Fisher Scientific, Waltham, MA, USA) to remove organic compounds. The solutions obtained were analysed by ion chromatography (IC‐Dionex DX120) under conductivity detection, by using an IonPac AG14 precolumn and an IonPac AS14 separation column (Thermo Fisher Scientific) at 35 °C, flow 1 mL min^−1^. Three replicates were performed for each biological treatment. Results were expressed as grams of nitrate per kilogram of fresh weight (g NO_3_^−^ kg^−1^ FW). These values were critically compared to the maximum nitrate limits established for leafy vegetables by European Commission Regulations No. (EU) 2023/915^4^ ranging from 6 to 7 g kg^−1^ FW, depending on the season and specific product.

### Vitamin C analysis

Vitamin C content, defined as the sum of l‐ascorbic acid (AA) and dehydroascorbic acid (DHAA), was quantified using a high‐performance liquid chromatography (HPLC) method as described by Cefola *et al*.^14^ A 5 g sample of fresh tissue was homogenized with 25 mL of 3% metaphosphoric acid. The homogenate was centrifuged at 10 000 × *g* for 15 min at 4 °C, and the supernatant was filtered through a 0.45 μm nylon syringe filter. Separation was achieved on a C18 column (250 mm × 4.6 mm, 5 μm particle size) with a mobile phase of 0.1% sulphuric acid (H_2_SO_4_) at a flow rate of 0.8 mL min^−1^. Detection was performed at 254 nm. Quantification was based on external standards of AA and DHAA. All analyses were performed in triplicate. Results were expressed as grams per kilogram of fresh weight (g kg^−1^ FW). For each level of nitrogen fertilization, individual degradation curves of total vitamin C, AA, and DHAA over time were fitted with a first‐order kinetic model (Eqn 1):

(1) $$Y=A_{0}\exp\left(-\mathrm{kt}\right)$$

where *Y* represents the concentration of the vitamin C (in g kg^−1^) at time *t* (in days), *A*_0_ is the initial concentration (in g kg^−1^) at the start of storage (*t* = 0), and *k* is the apparent degradation rate constant (in d^−1^), estimated by the slope of the linearized form of the curve.

### Microbial analysis

Total mesophilic bacteria, yeast and mould counts were analysed as indicators of general hygiene, microbial spoilage, and quality degradation rather than direct pathogen‐related food safety risks. For microbiological enumeration, 5 g of rocket leaves from each sample were aseptically weighed and homogenized with 45 mL of sterile 0.1% peptone water using a stomacher (BagMixer 400; Interscience, France) for 1 min. Serial decimal dilutions were prepared.

Mesophilic aerobic bacteria enumeration was performed using plate count agar (PCA; Oxoid, Basingstoke, UK). Plates were incubated at 35 °C for 48 h, following the recommended international standard ISO 4833‐1:2013^15^ for enumeration of microorganisms.

Similarly, for yeasts and moulds, potato dextrose agar (PDA; Oxoid, Basingstoke, UK) supplemented with chloramphenicol (0.1 g L^−1^) was used. Plates were incubated at 25 °C for 5 days, adhering to the recommended international standard ISO 21527‐2:2008^16^ for enumeration of yeasts and moulds.

The microbial colonies were counted from three replicates of each biological treatment, and results were expressed as log_10_ colony forming units per gram (log CFU g^−1^). Microbial growth for all six nitrogen fertilizer levels was modelled using the lag‐exponential model (Eqn 2):

(2) $$Y=b_{\max}-\ln\left[1+\left(10^{\left(b_{\max}-b_{0}\right)}-1\right)\left(-k\right)t\right]$$

where *Y* is the cell concentration at time *t* (in log CFU g^−1^), *b*_0_ is the initial cell concentration (in log CFU g^−1^), *b*_max_ is the maximum cell concentration achieved (in log CFU g^−1^), and *k* is the microbial growth rate (in d^−1^). In cases where a distinct lag phase was absent, a simplified exponential model was used.

### Chlorophyll analysis

For chlorophyll analysis, 1 g of frozen rocket leaf samples was accurately weighed and extracted in subsequent portions with a total of 15 mL of 100% methanol. Samples were kept in the dark at 4 °C for 24 h to ensure complete pigment extraction, until no visible green pigment remained in the leaf tissue. The mixtures of the methanolic extracts were then measured through a spectrophotometer with specific wavelengths of 666 nm (chlorophyll *a* (Chl *a*)), 653 nm (chlorophyll *b* (Chl *b*)) and 470 nm (carotenoids) (Shimadzu UV‐1800; Shimadzu, Kyoto, Japan). The concentration of Chl *a*, Chl *b* and total chlorophyll (Total Chl) was determined by the following equations as in van Loey *et al*.,^17^ and the results were presented in the form of milligrams per 100 g of fresh weight (mg 100 g^−1^ FW). Each analysis was done thrice. The kinetics of chlorophyll degradation was studied accurately by non‐linear exponential decay function that was developed to explain the complex interaction between nitrogen supply and after harvest shelf‐life. The need to explain the variations in both nitrogen‐dependent initial chlorophyll concentration and a variable degradation rate dependent on a time exponent led to the development of this model as it is a complex biological senescence. The generalized form of the model employed was (Eqn 3):

(3) $$C\left(t\right)=\left(C_{1}N+C_{2}\right)\;\exp\left[-\left(k_{1}N+k_{2}\right)\;t^{n}\right]$$

where *C*(*t*) is the concentration of chlorophyll (in mg kg^−1^ FW) at a particular time, *t* (in days), after harvest; *N* represents the amount of nitrogen supplied (in mol L^−1^) in the growth of the plant. The parameters *C*_1_ and *C*_2_ show the linear correlation of initial chlorophyll concentration to the availability of nitrogen; the effect of nitrogen as a key involved element during the biosynthesis of chlorophyll.^18^ The parameters *k*_1_ and *k*_2_ measure the effect of nitrogen on the rate constant of degradation of chlorophyll, which captures metabolic reactions to the state of nitrogen. The exponent ‘*n*’ is the time exponent, which is a very important factor that enables the model to embrace non‐first‐order kinetics of degradation, which reflects the accelerating or decelerating degradation kinetics of degradation processes during the storage period. Parameters *C*_1_, *C*_2_, *k*_1_, *k*_2_, and *n* were estimated simultaneously using non‐linear least squares regression. This approach minimizes the sum of squared residuals between the observed and predicted chlorophyll concentrations across all experimental treatments and time points. The iterative fitting process for the time exponent *n* was optimized to achieve the best possible fit to the experimental data.

### Texture analysis

Rocket leaf texture was evaluated on 5 g samples using a texturometer (TA.XT plus; Stable Micro Systems, Godalming, UK), equipped with a ‘micro Kramer‐like shear cell’ composed of five stainless‐steel blades. The samples were placed in the cell, and the blades extruded the leaves with the crosshead operating at a constant speed of 50 mm min^−1^. Texture was measured as the maximum peak force (in newtons, N) required to shear the leaves. For each treatment and time point, three biological replicates were measured. A linear equation (Eqn 4) with slight modifications^19^ was found to successfully describe texture changes as a function of storage time (*t*):

(4) $$Y=F_{0}-k_{\text{texture}}t$$

where *Y* is the maximum force (in newtons) required to cut the rocket leaves at time *t*, *F*_0_ is the initial maximum force (in newtons) at *t* = 0, and *k*_texture_ is the apparent texture degradation rate constant (in d^−1^).

### Sensory evaluation

The overall acceptability of rocket leaves was evaluated by ten trained assessors (five male and five females, between 25 and 45 years), on the overall acceptability of the leaves. The selection and training of these assessors were based on ISO 8586:2012^20^ criteria, that is, they had the capacity to obtain and report consistent sensory characteristics of fresh produce. The panel rated the overall acceptability according to the visual appearance change during the entire period of the storage process with a structured five‐point hedonic scale. A score 5 represented very high quality in that they were uniform dark green leaves of fresh and turgid appearance. Good quality was considered a score of 4 and the fresh leaves had slight loss of turgidity. The score of 3 was a good indication of high loss of turgidity and color and the commencement of wilting, which was still acceptable as marketable limit. The score of 2 indicated low quality, high senescence, wrinkled and yellowish blades, which were not acceptable in the market. Lastly, a very poor quality scored 1, where the leaves were spoiled and had severe cases of wilting, severe yellowing and decay. Shelf‐life was identified to be the days needed to achieve the overall acceptability score to the marketability limit, which is a score of 3.^21^ The overall acceptability data were modelled using a first‐order equation (Eqn 5) to explain the sensory deterioration with age occurring as a result of physiological degradation:

(5) $$Y=A_{0}\exp\left(-\mathrm{kt}\right)$$

where *Y* is the cumulative acceptability score at time *t*, *A*_0_ is the starting score (maximum = 5 at *t* = 0), *k* is the rate constant of degradation (unique to each nitrogen treatment), *t* is time in days.

Shelf‐life is defined as the time when the sensory score drops to 3, the marketability threshold (Eqn 6):

(6) $$t_{\text{shelf}-\text{life}}=\frac{\ln\left(A_{0}/3\right)}{k}$$

### Statistical analysis and kinetic modelling

All experiments followed a randomized block design with three independent biological replicates per treatment. Each analytical procedure (nitrate, vitamin C, microbial, chlorophyll, texture) was performed with at least three technical replicates.

Primary model parameters (initial concentrations *A*_0_ or *b*_0_, maximum concentrations *b*_max_, and degradation/growth rate constants *k*) were estimated by non‐linear regression analysis.

Secondary models, aimed at quantifying the relationship between nitrogen fertilization level (*N*) and the primary model parameters, were developed using regression analysis. The best‐fitting equations for *A*_*0*_ and *k* as a function of *N* were selected based on the highest coefficient of determination (*R* ^2^) and the lowest residual sum of squares (SS_r_) between experimental and predicted values. The general forms of these secondary models were (Eqns 7 and 8):

(7) $$A_{0}=A_{0a}\ln\left(N\right)+A_{0b}$$

(8) $$k=k_{a}\left(N\right)+k_{b}$$

The statistical analyses, including analysis of variance (ANOVA) and regression, were performed using Statgraphics Centurion XVI (StatPoint Technologies, Inc., Warrenton, VA, USA). The *R* ^2^, root mean square error (RMSE) and inspection of plots of the residual values were used to determine model adequacy by the lack of trend. Relative error (RE) was also computed to determine the predictive power of the models and the value between ±20% is satisfactory. Further rigor of methods employed was by adhering to the suggestions on the experimental design^22^ and non‐linear regression of biological data. Criterion of reproducibility and repeatability was taken into consideration in line with ISO 5725‐2:2025.^23^ Model validation was conducted by regressing observed values on model predictions using the relationship Observed = *a* + *b* × Predicted. We report the intercept and slope (with standard error (SE)), validation *R* ^2^, RMSE, and the tests for *a* = 0 and *b* = 1 to evaluate potential bias.

## RESULTS AND DISCUSSION

### Nitrate levels

Factors such as nitrogen fertilization have strongly influenced accumulation of nitrates in leafy vegetables. In the present study, nitrate concentration in rocket leaves increased nitrogen supply increase, which was evident at both the initial (February) and the final (May) harvest, as shown in Table 1.

During the last harvest, the nitrate content of T_1_ was 3.62 g kg^−1^ FW, and T_6_ recorded the highest 12.14 g kg^−1^ FW. These values affirm the importance of fertilization management in promoting the quality of products as well as consumer safety. The European Commission Regulations No. (EU) 2023/915,^4^ states that the maximum admissible nitrate level of rocket leaves is usually between 6 and 7 g kg^−1^ FW depending on the time of the year. Particularly, the harvesting limit of rocket that has been harvested between 1 October and 31 March is 7 g kg^−1^ FW and during the period 1 April to 30 September it is 6 g kg^−1^ FW. These are the periods when our harvests were made. In our experiment, the values of nitrate (last harvest) in treatment T_5_ (11.07 g kg^−1^ FW) and T_6_ (12.14 g kg^−1^ FW) were well above these legal limits, which caused serious potential safety concerns, and made these two treatments unsuitable to be commercialized in the European Union (EU), although treatment T_4_ (8.82 g kg^−1^ FW) was also not below the limit.^4^ In addition, it is interesting to note that the level of nitrates was also usually higher in the final harvest than in the initial harvest in treatment T_2_ through T_6_ (Table 1). This might be explained by the fact that the level of light and ambient temperatures increased during the last months (April–May) as we can see in Fig. 1, which showed that mean photosynthetically active radiation (PAR) and temperature increased starting from the beginning of March, the former doubling the mean value of the previous months and the temperature increasing linearly from March to May.

**Figure 1:**
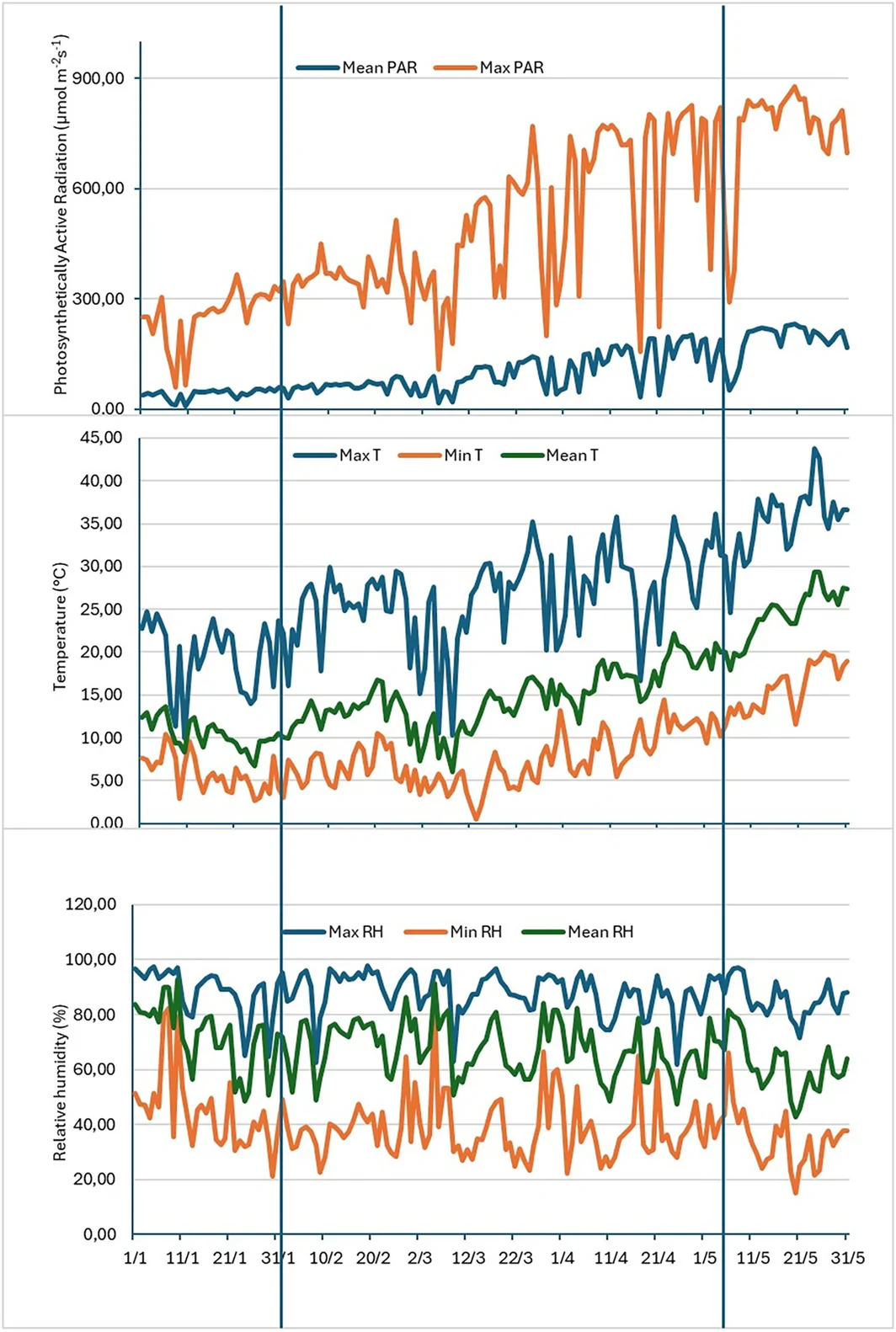
Climate parameters (mean and maximum photosynthetically active radiation (PAR); minimum, maximum and mean air temperature (*T*); and minimum, maximum and mean relative humidity (RH)) recorded in the glasshouse during the entire trial from January to May 2022. Vertical lines indicate the time of the first and final harvests.

Which might have an effect on the nitrogen uptake and metabolism of plants, but these related physiological parameters were not measured in this study, in successive cuttings the plants were repeatedly cut off, and had to quickly regenerate their leaf canopy with a full and vigorous root system. In this case, the newly produced leaves become efficient nitrogen sinks but do not have much time to metabolize the nitrogen they have taken up before the subsequent harvest. This physiological unbalance may cause an increase in the amount of accumulation of nitrates in subsequent cuttings especially with a high amount of nitrogen. Furthermore, the peat based substrate employed in the system is not totally inert and will hold some portion of the nutrient supplied to it, leading to a slow build‐up of nitrogen supply over time even though the NS is open loop managed.

This is a common problem with nitrate management in leafy greens due to seasonal changes.^24^ Remarkably, the treatments with moderate nitrogen supply (T_1_–T_3_, equivalent to 0.005–0.009 mol L^−1^ nitrogen at last harvest, respectively) were within the limits of the law (T_1_ = 3.62, T_2_ = 5.12, T_3_ = 8.52 g kg^−1^ FW at last harvest) and even contained higher vitamin C levels (as seen in the section Vitamin C degradation kinetics), indicating that moderate nitrogen management can be used to maximize quality and safety. The result is in agreement with the previous reports in leafy vegetables where high nitrogen fertilization enhanced nitrate concentration and decreased antioxidant capacity.^11^, ^25^, ^26^ In addition, levels of nitrate were found to be mostly negatively related to levels of AA in our research, especially at elevated nitrogen levels and this is an indication of a potential competitive relationship between nitrate assimilation and ascorbate metabolism.

Correlation studies in terms of treatment means (T_1_–T_6_) of vitamin C and nitrate concentration at initial and final harvest based on three replicate values of each parameter are presented in (Fig. 2). In the initial harvest, the Pearson correlation between nitrate concentration and vitamin C was strong with a negative value of *r* (*r* = −0.889) and the *t*‐value (*t* = −3.88) was significant (*P* = 0.018). This inverse relationship was also amplified at the final harvest, where the relationship was very strong with negative correlation (*r* = −0.981, *n* = 6) and significantly so (*t* = −10.06, *P* = 0.001). These findings point to the fact that with the increase of nitrate level during the growth period, the drop of vitamin C level increased with a higher rate, which is indicative of a progressive inhibition of ascorbate metabolism with an increase in nitrogen status. This is in line with the recent observations that high nitrogen fertilization enhances storage of nitrates and decreases vitamin C and antioxidant power in leafy vegetables.^27^, ^28^ The same nitrate reduction trade‐offs have been observed between glasshouse cultivated leafy greens and various vegetable species under different fertilization regimes.^27^, ^29^ Taken together, the findings indicate that there is a significant and biologically significant negative association between nitrate content and vitamin C level at different harvest periods supporting the overall findings that too much nitrogen may negatively influence nutritional quality in leafy vegetables. This supports earlier findings that nitrate reductase activity and AA biosynthesis share overlapping metabolic pathways and can be affected by the redox status of the plant, which is influenced by nitrogen availability.^30^, ^31^ The high nitrate levels observed at elevated nitrogen supplies clearly indicate a critical safety concern that must be balanced with other quality attributes and yield optimization.

**Figure 2:**
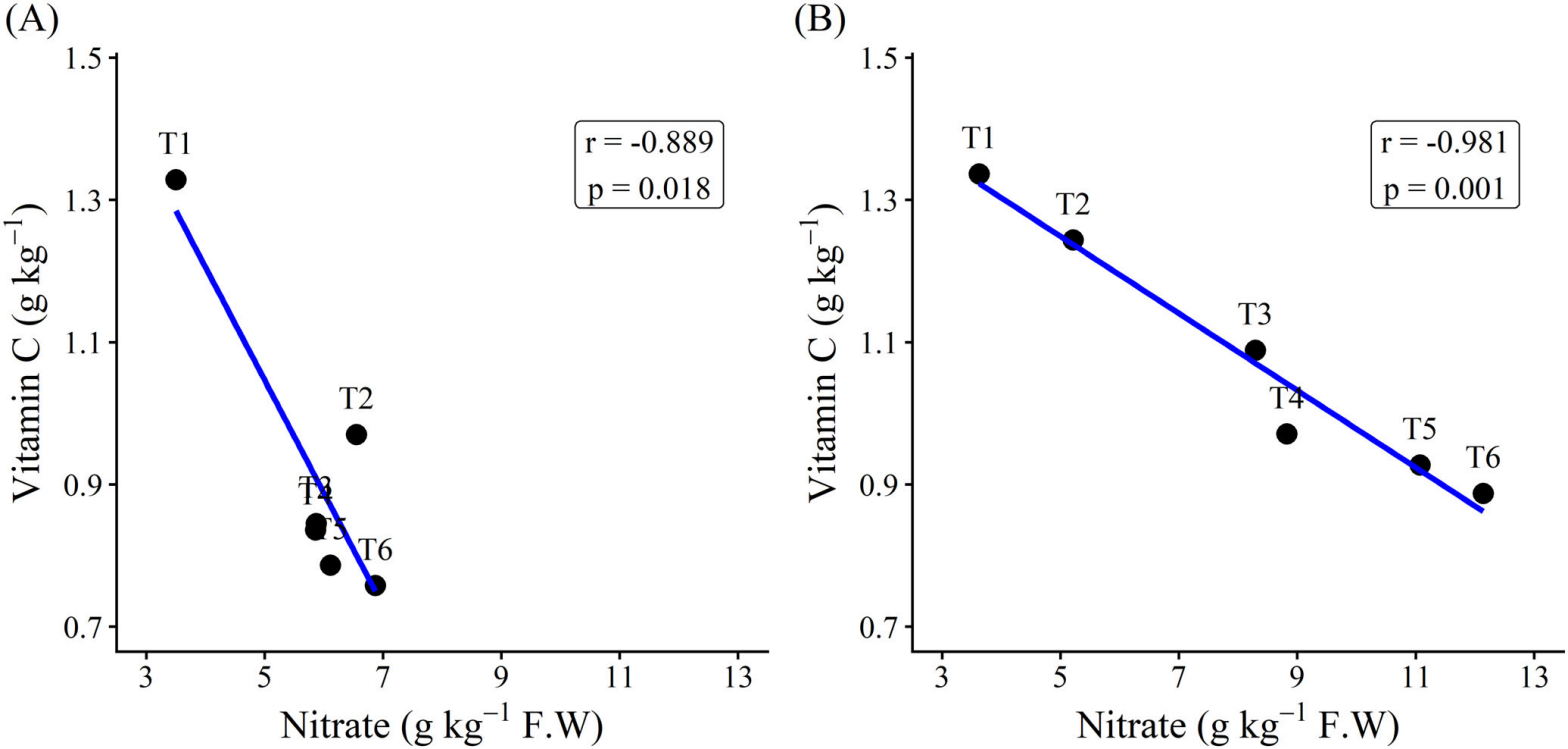
Relationship between nitrate (g kg^−1^ fresh weight (FW)) *x*‐axis and vitamin C content (g kg^−1^ FW) *y*‐axis of rocket leaves grown at six nitrogen fertilizer levels (T_1_–T_6_) at first harvest (A) and last harvest (B). Data points represent the treatment means calculated from three replicates (*n* = 3). Solid line represents linear regression fit. Pearson correlation analysis showed a significant negative relationship at both harvests (first harvest: *r* = −0.889, *P* = 0.018; last harvest: *r* = −0.981, *P* = 0.001).

### Vitamin C degradation kinetics

Vitamin C (AA + DHAA) plays a critical role in the nutritional value of fresh rocket leaves but is also highly sensitive to storage conditions. In this study, vitamin C degradation followed a first‐order kinetic model (Eqn 1) (*R* ^2^ > 0.90), consistent with previous reports on leafy vegetables.^11^, ^14^ Figure 3 clearly illustrates the time‐dependent degradation of total vitamin C for all six treatments, with solid markers representing experimental data and dotted lines showing the fitted first‐order model.

**Figure 3:**
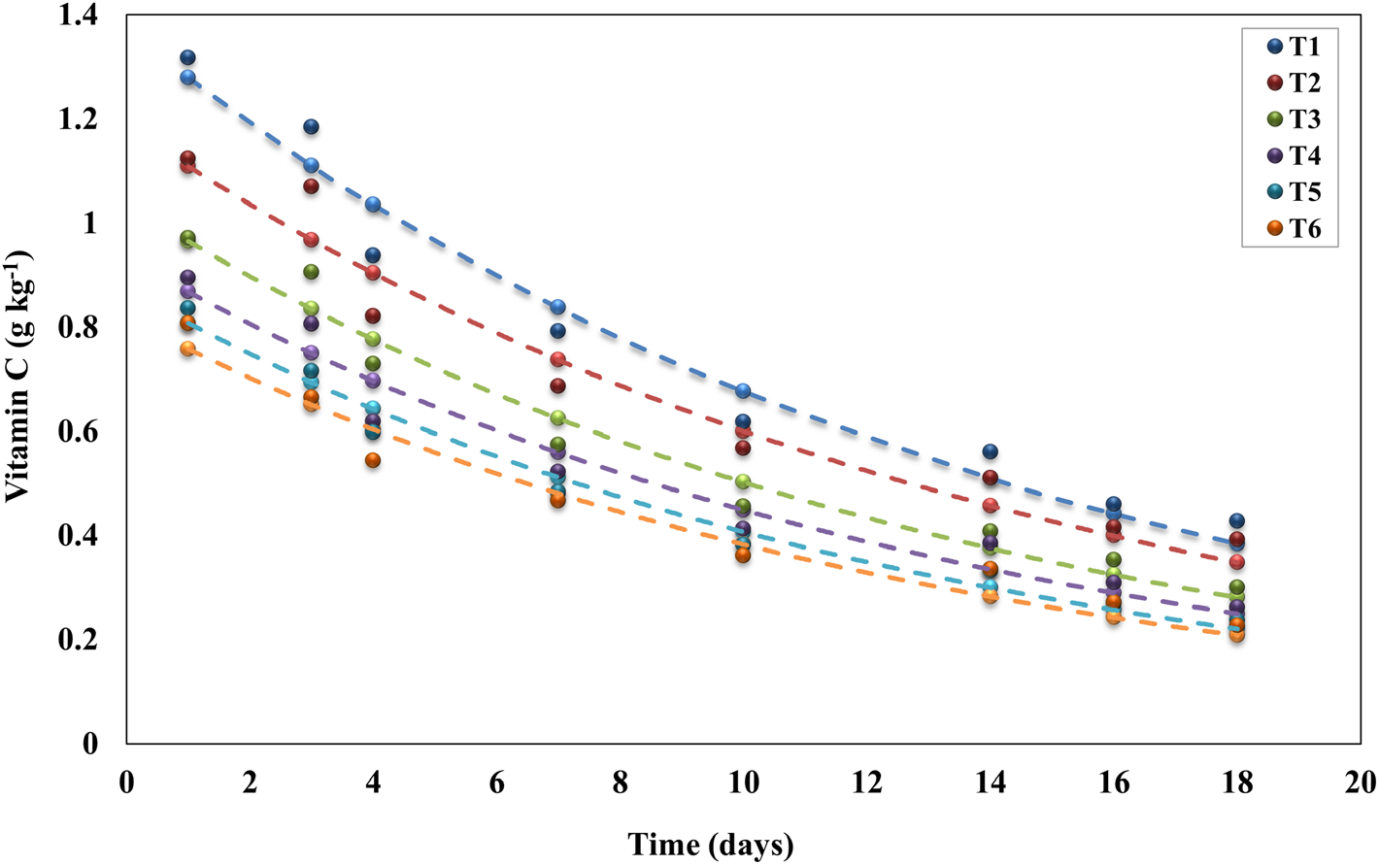
Vitamin C (g kg^−1^) degradation in all six treatments (T_1_–T_6_) *y*‐axis of rocket leaves during an 18‐days storage period at 5 °C *x*‐axis. Solid filled markers (●) correspond to experimental values with the individual treatments distinguished by distinct colour‐coded markers (see figure legend). Each experimental data point represents the grand mean (n=15) derived from five independent harvest experiments, with three replicates analysed per sampling interval. Dotted lines (‐‐‐) represent the fitted model values obtained through first order kinetic model (Eqn (1): $Y=A_{0}\exp\left(-\mathrm{kt}\right))$.

Initial concentrations of total vitamin C at harvest (*A*_0_, Table 2) varied significantly among treatments, with the highest noted for treatment T_1_ (1.37 g kg^−1^ FW), followed by T_2_ (1.18 g kg^−1^ FW). The lowest initial concentration was observed in T_6_ (0.81 g kg^−1^ FW). This inverse relationship between initial vitamin C content and nitrogen fertilization level is in agreement with previous work,^28^, ^32^ who reported decreased vitamin C with increasing nitrogen doses in various leafy greens. This reduction in initial vitamin C at higher nitrogen levels might be attributed to a dilution effect due to increased vegetative growth and larger leaves, or potentially to a metabolic shift favouring nitrogen assimilation over vitamin C synthesis.

**Table 2:** Kinetic parameters degradation rate constant *k* (day^−1^), and initial values *A*_0_ (g kg^−1^) for vitamin C, l‐ascorbic acid (AA) and dehydroascorbic acid (DHAA), as calculated by using Eqn (1); $Y=A_{0}\exp\left(-\mathrm{kt}\right)$, *n* = 1, with relative statistics

Treatment	Quality attribute		Estimate	Standard error	Confidence limits	*R* ^2^
T_1_	Vitamin C	*A*_0_	1.37	0.05	1.23–1.51	0.99
		*k*	0.07	0.006	0.05–0.08	
	AA	*A*_0_	1.04	0.05	0.90–1.18	0.92
		*k*	0.06	0.008	0.04–0.07	
	DHAA	*A*_0_	0.30	0.03	0.22–0.38	0.83
		*k*	0.08	0.01	0.04–0.12	
T_2_	Vitamin C	*A*_0_	1.18	0.05	1.05–1.32	0.95
		*k*	0.06	0.007	0.05–0.08	
	AA	*A*_0_	0.98	0.05	0.84–1.11	0.93
		*k*	0.06	0.008	0.04–0.08	
	DHAA	*A*_0_	0.19	0.009	0.17–0.22	0.96
		*k*	0.08	0.007	0.06–0.10	
T_3_	Vitamin C	*A*_0_	1.03	0.04	0.93–1.14	0.96
		*k*	0.07	0.006	0.05–0.08	
	AA	*A*_0_	0.85	0.04	0.75–0.95	0.95
		*k*	0.06	0.007	0.05–0.08	
	DHAA	*A*_0_	0.14	0.006	0.12–0.15	0.96
		*k*	0.06	0.007	0.05–0.08	
T_4_	Vitamin C	*A*_0_	0.93	0.04	0.82–1.04	0.95
		*k*	0.07	0.007	0.05–0.09	
	AA	*A*_0_	0.77	0.04	0.68–0.87	0.94
		*k*	0.07	0.008	0.05–0.08	
	DHAA	*A*_0_	0.15	0.01	0.11–0.19	0.82
		*k*	0.08	0.01	0.04–0.13	
T_5_	Vitamin C	*A*_0_	0.87	0.02	0.80–0.94	0.98
		*k*	0.07	0.005	0.06–0.08	
	AA	*A*_0_	0.73	0.02	0.66–0.79	0.97
		*k*	0.07	0.005	0.06–0.08	
	DHAA	*A*_0_	0.13	0.01	0.10–0.17	0.82
		*k*	0.08	0.01	0.04–0.13	
T_6_	Vitamin C	*A*_0_	0.81	0.03	0.72–0.90	0.96
		*k*	0.07	0.007	0.05–0.09	
	AA	*A*_0_	0.67	0.02	0.60–0.74	0.96
		*k*	0.07	0.006	0.0–0.08	
	DHAA	*A*_0_	0.15	0.02	0.10–0.19	0.82
		*k*	0.09	0.002	0.04–0.15	

*Note*: Fitted parameter values ± 95% confidence intervals of the first order model – regression analysis.

Across storage, vitamin C content declined more rapidly at higher nitrogen levels. Treatments T_1_ and T_2_ showed the slowest degradation rates (*k* = 0.07 and 0.06 d^−1^, respectively, for total vitamin C, Table 2), whereas T_5_ and T_6_ exhibited similar but slightly higher degradation rates (*k* = 0.07 d^−1^). Treatments T_5_ and T_6_ showed higher degradation rate of AA and DHAA individually (*k* = 0.07–0.09), which is more pronounced for DHAA. This suggests that excessive nitrogen supply, potentially by increasing tissue water content and respiration, accelerates oxidative degradation of AA, in agreement with De Corato,^33^ who demonstrated that higher nitrate content correlates with faster ascorbate decline in leafy greens.

DHAA also degraded faster than AA (e.g., *k* = 0.08 for T_1_ DHAA *versus k* = 0.06 for T_1_ AA, Table 2), confirming that AA degradation directly contributed to the total vitamin C loss. The amount of DHAA made a minimal contribution to the total vitamin C content (less than 15% for T_6_), indicating that AA was the dominant form. The faster degradation of DHAA, as supported by higher *k* values in Table 2, could be attributed to its irreversible conversion into diketogulonic acid, which is an inactive form, thus contributing to the overall loss of vitamin C. However, the current analysis applied independent first‐order fits for AA and DHAA. Given the metabolic pathway AA → DHAA inactive products, future studies should consider using consecutive reaction models (e.g., as described by Katsch *et al*.^34^) to better describe these complex transformations and provide a more mechanistic understanding of vitamin C degradation.

Notably, the retention of vitamin C after 14 days of storage (which was commonly regarded as a commercial shelf‐life of fresh‐cut rocket) was greater than 70% in T_1_ and T_2_, but much lower (39.78% and 41.49%) in T_5_ and T_6_, again demonstrating the unhealthy influence of excessive nitrogen fertilization on nutritional value throughout storage. The vitamin C values obtained in this study (between 0.81 and 1.37 g kg^−1^ FW) are significantly greater than the literature studies, for example, Marchioni *et al*.^35^ only reported very low nutrient amounts of 29.67 μg g^−1^ FW (or 0.029 g^−1^ FW) which might be because this research was done on a different cultivar of rocket, in different growing conditions or the method used of analysis.

It is important to develop secondary models to predict by relating primary kinetic parameters to nitrogen levels. Figure 4 illustrates the curve fitting of the degradation rate constant ‘*k*’ and the initial concentration ‘*A*_0_’ for AA as a function of nitrogen fertilizer levels, allowing for the construction of secondary model equations (Eqns 7 and 8). These secondary model parameters (*A*_0a_, *A*_0b_, *k*_a_, *k*_b_ for vitamin C, AA, and DHAA) are presented in Table 3. The obtained secondary model (Eqn (9)), relating vitamin C concentration directly to storage time and nitrogen fertilizer level, showed good agreement with observed values, with *R* ^2^ values of 0.92 for total vitamin C, 0.89 for AA, and 0.77 for DHAA (Table 3). Equation (9) integrates the secondary models for *A*_0_ (Eqn 7) and *k* (Eqn 8) into the primary first‐order degradation model (Eqn 1), allowing direct prediction of vitamin C concentration based on both storage time and nitrogen fertilizer level. The model was further validated with RE values remaining within the acceptable ±20% limit. Figure 5 visually confirms the good fit between the prediction model results (Eqn 9) and the experimental AA values across all treatments and storage intervals. This demonstrates the robust predictive capability of the developed models for vitamin C degradation as a function of preharvest nitrogen fertilization. The vitamin C models were validated by regressing observed concentrations on model predictions using Observed = *a* + *b* ×Predicted. Important results are shown in Table 3. The *R* ^2^ values ranged between 0.776 (DHAA) and 0.920 for (vitamin C) and this shows that there was a high correlation between the observed and predicted values. The non‐significant test comparing *a* = 0, *b* = 1 shows that the slope and intercept of the regression are statistically equivalent to the hypothetical values (no systematic offset and unit slope), which shows that the model predictions are not biased.

(9) $$Y=A_{0a}\ln\left(N\right)+A_{0b}\exp\left[-\left(k_{a}\ln\left(N\right)+k_{b}\right)t\right]$$

**Figure 4:**
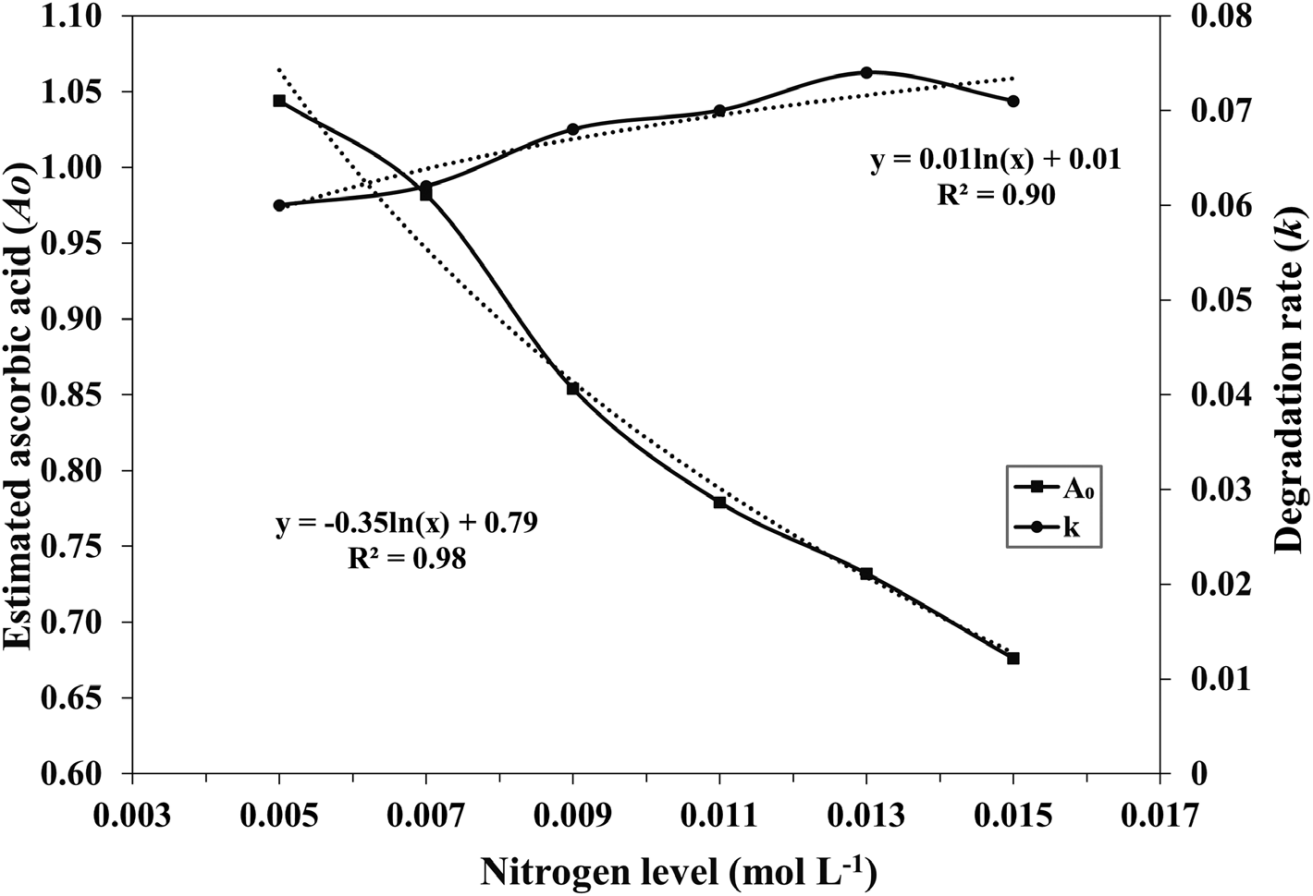
Curve fitting of primary kinetic parameters for ascorbic acid degradation in rocket leaves grown at six nitrogen fertilizer levels ranging from 0.005 to 0.015 mol L^−1^. The graph shows degradation rate constant *k* (●; d^−1^), and estimated ascorbic acid values *A*_0_ (■; g kg^−1^) at initial day of storage calculated by using Eqn (1) (*Y* = *A*_0_exp(−*kt*)) as a function of nitrogen fertilization (*x*‐axis) in nutrient solution. Solid lines showed secondary regression model used to derive the secondary kinetic model parameters (*A*_0*a*_, *A*_0*b*_ and *k*_*a*_, *k*_*b*_).

**Table 3:** Secondary model parameters for vitamin C, dehydroascorbic acid, and ascorbic acid, as calculated by using Eqn (9), where $A_{0}=A_{0a}\ln\left(N\right)+A_{0b}$ and $k=k_{a}\left(N\right)+k_{b}$ and regression‐based validation metrics obtained from the linear regression of observed *versus* predicted values (Observed = *a* + *b* × Predicted)

Secondary model parameters					
Quality attributes	*A*_0a_	*A*_0b_	*k*_*a*_	*k*_*b*_	*R* ^2^
Vitamin C	−0.50	3.47	0.006	0.04	0.92
Dehydroascorbic acid	−0.13	0.83	0.03	−0.05	0.77
Ascorbic acid	−0.35	0.79	0.01	0.01	0.89

Validation metrics (Observed *versus* Predicted regression)				
Attribute	Intercept (SE); *P* (*a* = 0)	Slope (SE); *P* (*b* = 1)	*R* ^2^	RMSE
Vitamin C	0.96 (1.58); *P* = 0.54	0.99 (0.03); *P* = 0.69	0.92	7.79
Dehydroascorbic acid	0.56 (0.48); *P* = 0.24	0.95 (0.04); *P* = 0.31	0.77	2.96
Ascorbic acid	0.85 (1.55); *P* = 0.58	0.98 (0.03); *P* = 0.61	0.89	7.18

*Note*: *R* ^2^, coefficient of determination; RMSE, root mean square error; SE, standard error.

**Figure 5:**
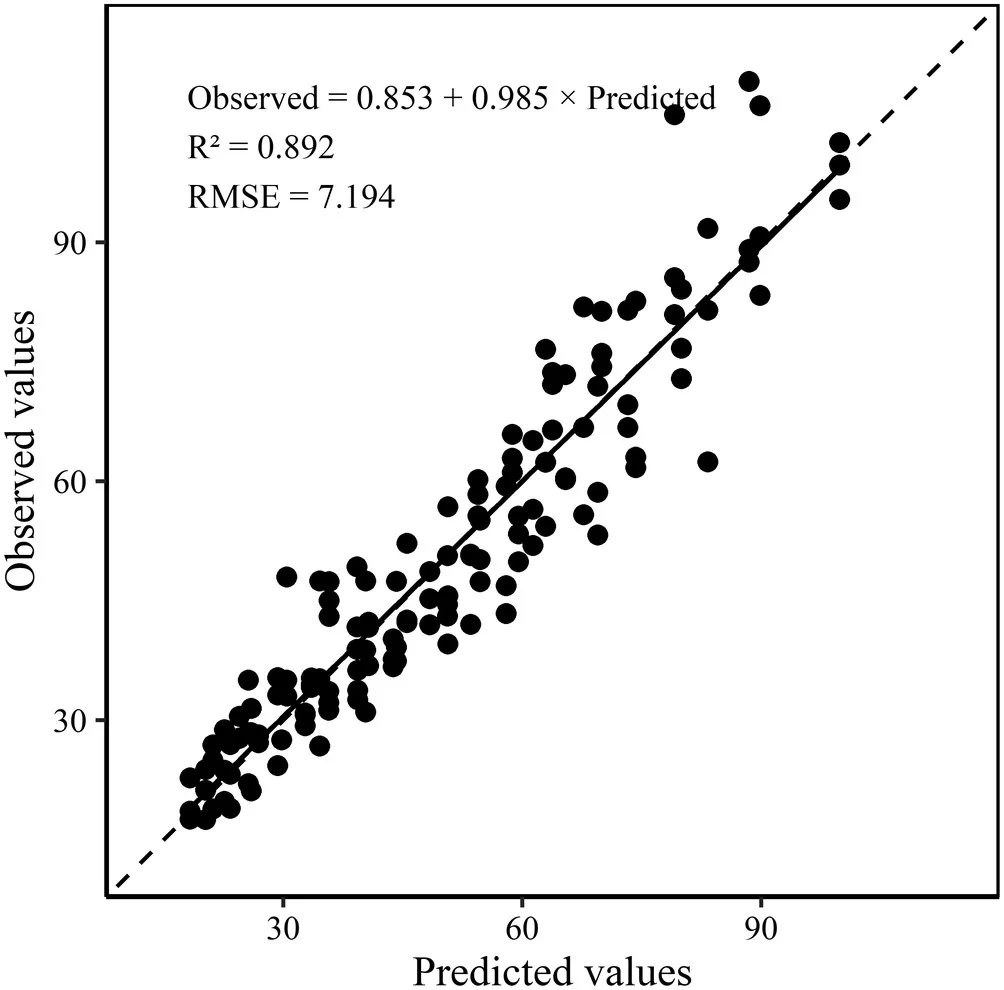
Correlation plot between predicted *versus* experimental ascorbic acid values for rocket leaves grown at six nitrogen fertilizer levels during an 18‐days storage period at 5 °C. Prediction model results calculated by using Eqn (9), parameters mentioned in Table 3 *versus* ascorbic acid experimental values for all treatments at all studied storage intervals. The line represents the fitted linear regression between the predicted and observed data points (including the coefficient of determination, *R* ^2^, and root mean square error, RMSE).

### Microbial load

Microbial growth is a major factor limiting the shelf‐life and safety of fresh‐cut leafy vegetables. Initial average microbial counts ranged from 2.70 to 4.80 log CFU g^−1^ for total mesophilic count, and from 2.50 to 4.60 log CFU g^−1^ for yeasts and moulds across the treatments. These values are typical for fresh‐cut produce at the point of packaging.^36^

Mesophilic bacteria count increased progressively during storage. While the microbial growth was ‘significantly influenced by storage duration’, a closer look at the data reveals that higher nitrogen supply treatments (T_5_ and T_6_) generally experienced faster microbial proliferation, reaching higher populations earlier. For example, is the mesophilic bacteria counts in treatments that had elevated nitrogen supply (T_5_ and T_6_) which started with the mean of about 4.5 log CFU g^−1^ and reached beyond 7 log CFU g^−1^ after the 14 days. However, T_1_ and T_2_ had a lower reading than the recommended spoilage level of 7 log CFU g^−1^ of fresh‐cut produce at 16 days of storage, implying that moderate levels of nitrogen slowed down the growth of microbes. The microbial growth was well fitted using the lag‐exponential model (*R* ^2^ = 0.84–0.96, Table 4), meaning that the model is appropriate to model the dynamics of microorganisms in leafy vegetables. Visual representation of this growth rate *k* within the mesophilic, yeast and mould counts of varying levels of nitrogen fertilizers is presented in Fig. 6(a). It presents a fascinating pattern as the degradation rate ‘*k*’ (in d^−1^) of total mesophilic count rose with the level of nitrogen, and it was observed that the value of 0.35 to 0.51 d^−1^ existed during 18 days at 5 °C. More importantly, the increase in the number of fungi (yeasts and moulds) at an increased nitrogen concentration (T_4_, T_5_ and T_6_) was much more rapid (*k* values of 1.42, 1.53 and 1.76 d^−1^) than the mesophilic counts, which showed that fungi were predominant in the spoilage process under such circumstances. This observation is in agreement with Spadafora *et al*.^37^ who reported that fungal populations increased faster than the total mesophilic populations in the course of storing rocket leaves. The derived secondary model (Eqn 1), which incorporated the nitrogen fertilizer levels in microbial growth rate increased the predictive abilities further.

(10) $$Y=b_{\max}-\ln\left[1+\left(10^{\left(b_{\max}-b_{0}\right)}-1\right)\left(-a\left(\mathrm{bN}\right)\right)t\right]$$

**Table 4:** Kinetic parameters of secondary model (Eqn (10)) predicting microbial growth as a function of nitrogen levels for rocket leaves stored at 5 °C for 18 days and regression‐based validation metrics obtained from the linear regression of observed *versus* predicted values (Observed = *a* + *b* × Predicted). Model‐fit coefficient of determination (*R* ^2^) refers to the secondary model; validation *R* ^2^, root mean square error (RMSE), and parameter tests refer to the regression diagnostics

Secondary model parameters					
Quality attributes	Estimated kinetic parameters				*R* ^2^
	*a* (d^−1^)	*b* (d^−1^)	*b*_max_ (log CFU g^−1^)	*b*_0_ (log CFU g^−1^)	
Total mesophilic count	0.24	0.003	4.59	3.01	0.84
Yeast and mould	0.42	0.001	4.79	2.71	0.96

Validation metrics (Observed *vs*. Predicted regression)				
Attribute	Intercept (SE); *P* (*a* = 0)	Slope (SE); *P* (*b* = 1)	*R* ^2^	RMSE
Total mesophilic count	−0.03 (0.14); *P* = 0.80	1.00 (0.04); *P* = 0.80	0.84	0.22
Yeast and mould	0.11 (0.08); *P* = 0.08	0.96 (0.01); *P* = 0.097	0.96	0.18

*Note*: *b*_0_, initial cell concentration; *b*_max_, maximum cell concentration; *k*, microbial growth rate (d^−1^) where *a* and *b* are parameters of *k*. CFU, colony forming unit; SE, standard error.

**Figure 6:**
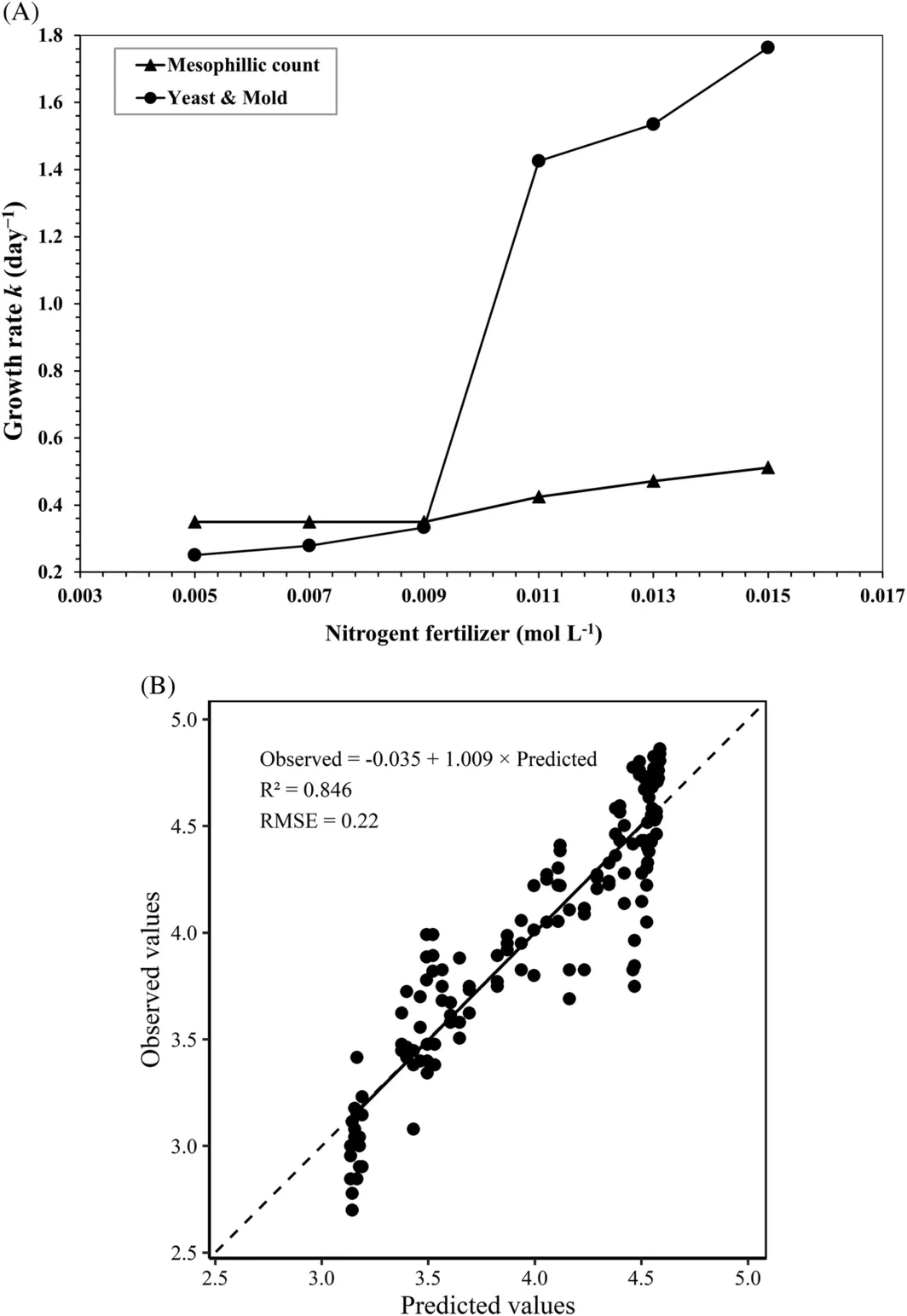
Microbial growth kinetics and secondary model prediction for rocket leaves (*Diplotaxis tenuifolia* L.) stored at 5 °C for 18 days. (a) Visual representation of difference between growth rate constant ‘*k*’ (d^−1^) *y*‐axis of mesophilic, yeast and moulds count for all six rocket leave samples grown at different nitrogen fertilizer levels (0.005–0.015 mol L^−1^) *x*‐axis. ‘*k*’ is primary model kinetic parameter calculated by a primary lag‐logistic model (Eqn 2). (b) Correlation plot comparing the experimental results of total mesophilic count *versus* predicted values calculated by secondary model Eqn (10) using the parameters mentioned in Table 2. The line represents the fitted linear regression between the predicted and observed data points (including the coefficient of determination, *R* ^2^, and root mean square error, RMSE).

Equation (10) represents the lag‐exponential model (Eqn 2) where the microbial growth rate ‘*k*’ is substituted by a secondary model that describes its dependence on nitrogen fertilizer level (*N*), allowing direct prediction of cell concentration based on time and nitrogen level. The parameters for this model are detailed in Table 4, showing good *R* ^2^ values (0.84 for mesophilic count, 0.96 for yeast and mould). Figure 6(b) illustrates the good fit between the predicted values from the secondary model (Eqn 10) and the experimental values for total mesophilic count. This strong predictive capability is valuable for assessing microbial safety risks linked to preharvest nitrogen management.

The observed faster microbial growth at higher nitrogen levels could be due to increased tissue turgidity and potentially higher surface exudates, which create a more favourable environment for microbial attachment and proliferation.^38^ Fungi generally have a wider pH tolerance, allowing them to thrive in more acidic conditions where many bacteria are inhibited,^39^ however, rocket leaves typically have a pH higher than pH 5. Our findings suggest that at higher nitrogen levels, the overall physiological changes in the leaves (e.g., increased moisture content, altered defensive compounds) may favour faster microbial proliferation, especially by fungi and yeasts. The microbial counts never attained the critical level of 7 log CFU g^−1^ across all treatments before sensory rejection, except in later stages for high nitrogen treatments.

In the case of microbial properties, the observed log counts were plotted as a regression of model projections (Observed = *a* + *b* × Predicted); the results are given in Table 4. The mesophilic count regression had high agreement (*R* ^2^ = 0.846) and did not have significant differences between intercept and slope values to the ideal values (intercept = 0, slope = 1; all *P* > 0.05) thus the predicted mesophilic counts were not systematically biased on the log scale.

### Chlorophyll degradation kinetics

Chlorophyll content, a primary determinant of visual quality and marketability in green leafy vegetables, progressively decreased across storage in all treatments. While initial Total Chl concentration at harvest was generally higher in treatments with increased nitrogen supply (e.g., T_6_: 221.0 ± 5.0 mg/kg^−1^ FW and T_2_: 184.0 ± 4.0 mg/kg^−1^ FW), consistent with observations by^40^ regarding a positive relationship between nitrogen fertilization and leaf colour, this initial advantage was offset by accelerated degradation during postharvest storage. This is likely due to amino acid abundance and increased plant surface area, augmenting net photosynthesis rates at higher nitrogen levels. First‐order kinetics: $C\left(t\right)=C_{0}\exp\left(-\mathrm{kt}\right)$ adequately described chlorophyll degradation for Chl *a*, Chl *b*, and Total Chl (*R* ^2^ > 0.91 for all fits), consistent with previous reports on rocket and spinach.^37^, ^39^, ^41^ The degradation rate constant (*k*) for total chlorophyll was significantly faster in high nitrogen treatments (e.g., T_5_ and T_6_: 0.08–0.09 ± 0.002 d^−1^) compared to lower nitrogen treatments (e.g., T_1_ and T_2_: 0.04–0.05 ± 0.001 d^−1^). Table 5 shows these individual *k*‐values of Chl *a*, Chl *b* and Total Chl of all the treatments. In particular, the Chl *a*, which is the most common form of chlorophyll, showed a greater degradation rate in all treatments in comparison to Chl *b*, which supports its reported instability during the postharvest storage, in particular at 5 °C.^39^ It was demonstrated that model Eqn (3) was created to describe the complicated process of chlorophyll degradation in rocket, which explicitly connected preharvest nitrogen fertilization with postharvest quality and shelf‐life. The last structure model that was used, including initial chlorophyll content and the rate of degradation *k* that depend on nitrogen:

(11) $$C\left(t\right)=\left(1647.6N+194.6\right)\exp\left[-\left(2.08N+0.029\right)t^{1.32}\right]$$

**Table 5:** Rate constants for degradation of chlorophyll *a* (Chl *a*), chlorophyll *b* (Chl *b*), and total chlorophyll (Total Chl) content, as calculated by using $C\left(t\right)=C_{0}\exp\left(-\mathrm{kt}\right)$

Treatment	Chl *a k* (d^−1^)	Chl *b k* (d^−1^)	Total Chl *k* (d^−1^)
T_1_	0.04	0.04	0.04
T_2_	0.04	0.03	0.04
T_3_	0.04	0.05	0.05
T_4_	0.05	0.05	0.05
T_5_	0.06	0.05	0.06
T_6_	0.06	0.06	0.06

The slope *C*_1_ = 1647.6 and *C*_2_ = 194.6 characterize the important linear curve of the initial chlorophyll concentration in response to increase in the amount of nitrogen supplied. This is also physiologically compatible, with nitrogen being an essential part of the chlorophyll molecule, having a direct effect on the development and photosynthetic capacity of chloroplasts.^18^, ^42^ Increased availability of nitrogen enhances increased biosynthesis of chlorophyll, consequently increasing the harvest leaf greenness, an important visual quality feature of leafy vegetables.

However, the parameters of the degradation rate *k*_1_ = 2.086 and *k*_2_ = 0.0294 indicate a duality in the effect of nitrogen both enhancing synthesis before the harvest and possibly acting as a catabolic modulator after the harvest. Of notable interest is the fitted time exponent *n* = 1.32. It is an indication of a non‐first‐order degradation rate, in which the rate of chlorophyll loss increases with time in storage. This acceleration can be typical of progressive senescence, which was brought about by growth‐old oxidative stress accumulation, increased enzymatic activity (e.g., chlorophyllase, Mg‐dechelatase) and elevated levels of reactive oxygen species (ROS) that break up the photosynthetic machinery.^43^ This exponent significantly enhances the predictive accuracy of the model particularly in the late stages of the storage where yellowing is visible. According to the model, the joint effects of nitrogen fertilization and storage time are able to explain the 85.2% of the observed variability in the chlorophyll concentration. Linear increase in chlorophyll with nitrogen may be considered valid on the range 0.005–0.015 mol L^−1^, but above this range, biosynthesis saturation or osmotic stress, which may cause chlorophyll content plateau or decrement could occur.^44^ Although, initial levels of chlorophyll increased with increase in nitrogen levels (T_5_ and T_6_), however, they were characterized by high rates of degradation thus causing rapid reduction in greenness. The phenomenon indicates that whereas high levels of nitrogen favour the synthesis of chlorophyll, it can also lead to a less stable photosynthetic complex or an increase in the activity of chlorophyll‐degrading enzymes (e.g., chlorophyllase) after harvest.^45^, ^46^

Chlorophyll loss is directly proportional to consumer rejection through yellowing and loss of freshness so treatments T_1_ and T_2_ showed an ability to retain marketable colour up to 14–16 days. While treatments T_5_ and T_6_ showed major yellowing and dropped below the acceptance limits in 10–12 days. This cosmetic degradation, influenced by a rapid chlorophyll degradation, directly affected the general sensory quality and shelf‐life of the produce as explained in the section Shelf‐life determination based on sensory evaluation where it is highlighted that the lower the level of nitrogen fertilizers are, the longer the coloured qualities are preserved.

### Texture degradation kinetics

The maximum cutting force which is known as leaf firmness is an important quality parameter that determines the marketability and acceptance of fresh‐cut rocket leaves by consumers. The original values of rupture force at harvest exhibited a definite tendency depending on the level of fertilization with nitrogen. Particularly, at the start of shear, the highest shear force was recorded in T_2_ (3.8 ± 0.1 N) and T_3_ (3.7 ± 0.1 N), whereas treatments with increased nitrogen, T_5_ (3.1 ± 0.1 N) and T_6_ (3.2 ± 0.1 N), would have a slightly low firmness at harvest. This note proves that although high nitrogen supply can cause vegetative growth, it frequently causes the development of more succulent tissues and with thinner cell walls, resulting in less structural robustness and firmness at harvest.^47^, ^48^

In postharvest storage, there was a significant reduction in leaf firmness over time in all the treatments. The degradation of the texture was characterized by a zero‐order kinetic law as given by Eqn (4) $Y=F_{0}-k_{\text{texture}}t$, which fitted well (*R* ^2^ = 0.85–0.92 across treatments). The apparent degradation rates (*k*_texture_) of firmness were dependent on the levels of nitrogen: the lowest degradation rates (T_1_ and T_2_) were 0.007 ± 0.001 and 0.008 ± 0.001 N d^−1^, respectively, whereas degradation rate was higher in T_5_ and T_6_ that were 0.011 ± 0.002 and 0.012 ± 0.002 N d^−1^. These results agree with earlier analysis of minimally processed leafy vegetables in which linear or zero‐order models are useful in explaining the loss of firmness.^19^, ^49^

On day 14 of storage, the firmness reduced by around 30–40% in T_1_ and T_2_ meaning that it retained the textural characteristics better. As compared to T_5_ and T_6_ treatments which had greater decrease in firmness that was more than 50% of starting values. All these findings add to highlight that although high nitrogen supply positively influences yield, it also affects tissue integrity and increases softening in postharvest, thereby reducing the shelf‐life due to textural quality. This has been proposed by increased metabolic activity and by an increase in the levels of cell wall‐degrading enzymes, including pectin methyl esterase and polygalacturonase, which can be stimulated when the plants grow rapidly.^43^

### Shelf‐life determination based on sensory evaluation

Data of overall sensory acceptability for each nitrogen treatment (T_1_–T_6_) was fitted in a first‐order degradation kinetic equation (Eqn 1): $Y=A_{0}\exp\left(-\mathrm{kt}\right)$. Findings (Table 6) also indicated that there is a linear relationship between the degree of nitrogen and the degradation rate constant (*k*) and increased concentrations of nitrogen produced higher values of *k*.

**Table 6:** Shelf‐life degradation kinetics and model performance metrics: Sensory degradation *versus* nitrogen level (Eqn (1); $Y=A_{0}\exp\left(-\mathrm{kt}\right)$)

Treatment	Degradation rate (*k*)	Residual sum of squares (SS_r_)	Total sum of squares (SS_t_)	Coefficient of determination (*R* ^2^)
T_1_	0.028	0.14	6.81	0.97
T_2_	0.033	0.21	6.74	0.96
T_3_	0.038	0.31	6.59	0.95
T_4_	0.045	0.42	6.48	0.93
T_5_	0.052	0.53	6.39	0.91
T_6_	0.065	0.64	6.28	0.89

To measure this relationship, a secondary linear regression model was published: *k* = 0.0072 + 0.00011*N*, where *N* is the level of nitrogen (in mol L^−1^). The fitted model, which is based on the latest squares (*R* ^2^ = 0.99), shows that 99% of the variability in the degradation rate constant is accounted by the concentration of nitrogen. The intercept (0.0072) indicates the degradation rate at zero nitrogen concentration and the slope (0.00011) indicates the rate at which degradation changes with a unit increase in nitrogen. The model was found to fit well with insignificant unexplained variance (SS_r_ = 2.70 × 10^−7^, total sum of squares (SS_t_) = 1.83 × 10^−4^) and has no trend in residuals that is treated as non‐linear and non‐homoscedastic. The slope is positive, which supports the existence of a statistically significant and positive correlation between the rate of sensory decline and nitrogen input, which is in agreement with physiological evidence of the presence of a nitrogen‐accelerated tissue senescence.^50^

Equation (6), $t_{\text{shelf}-\mathrm{lif}e}=\frac{\ln\left(A_{0}/3\right)}{k}$, was used to determine shelf‐life, direct prediction of shelf‐life is possible using nitrogen levels replacing *k*. The sensory quality of the lower nitrogen treatments (T_1_ and T_2_) was retained up to 17.8 days but higher nitrogen treatments (T_5_ and T_6_) decayed quickly and scored below acceptability in less than 7.6 days. This underscores the fact that the postharvest shelf‐life is greatly reduced when nitrogen is applied in high amounts as preharvest.

Despite of an extension of the overall storage time to 18 days in the case of kinetic modelling, the commercially viable shelf‐life of packaged rocket is generally 6–7 days. Predicted sensory scores at day 7 were 4.06 (T_1_), 3.94 (T_2_), 3.78 (T_3_), 3.58 (T_4_), 3.37 (T_5_), and 3.09 (T_6_). Compared to low nitrogen treatment (T_1_), high nitrogen leaves (T_6_) exhibited 24% reduced sensory quality on day 7, and moderate nitrogen concentrations (T_3_ and T_4_) exhibited 712% reduced quality. Notably, none of the treatments fell below the acceptability threshold (score 3), indicating that the standard signal of that reduced shelf‐life is more of a distinctly oriented decision than an actual philological end of commercial life, which can be influenced by a number of preharvest and postharvest elements. Already, only the leaves of T_5_ and T_6_ treatments were close to the marketability threshold, and T_1_ and T_2_ treatments still had a broader range of quality. Figure 7 shows the reasons that the overall acceptability is predicted to decrease in 18 days with an initial score of 5 across all treatments. The graph also visually supports the difference in the degradation rates: T_1_ (lowest nitrogen) had the slowest rate and had the scores above 3 over the period of 17.8 days. Comparatively, T_6_ (highest nitrogen) had the fastest decline as it dropped below the acceptability level of 3 at 7.6 days. At the tenth day, T_1_ scored 3.7, while T_6_ had already reduced to 2.5. This graphical evidence goes a long way to suggest that increased preharvest application of nitrogen results in a faster postharvest deterioration in sensory attributes and a much shorter shelf‐life, which is correlational to the past studies.^12^, ^51^

**Figure 7:**
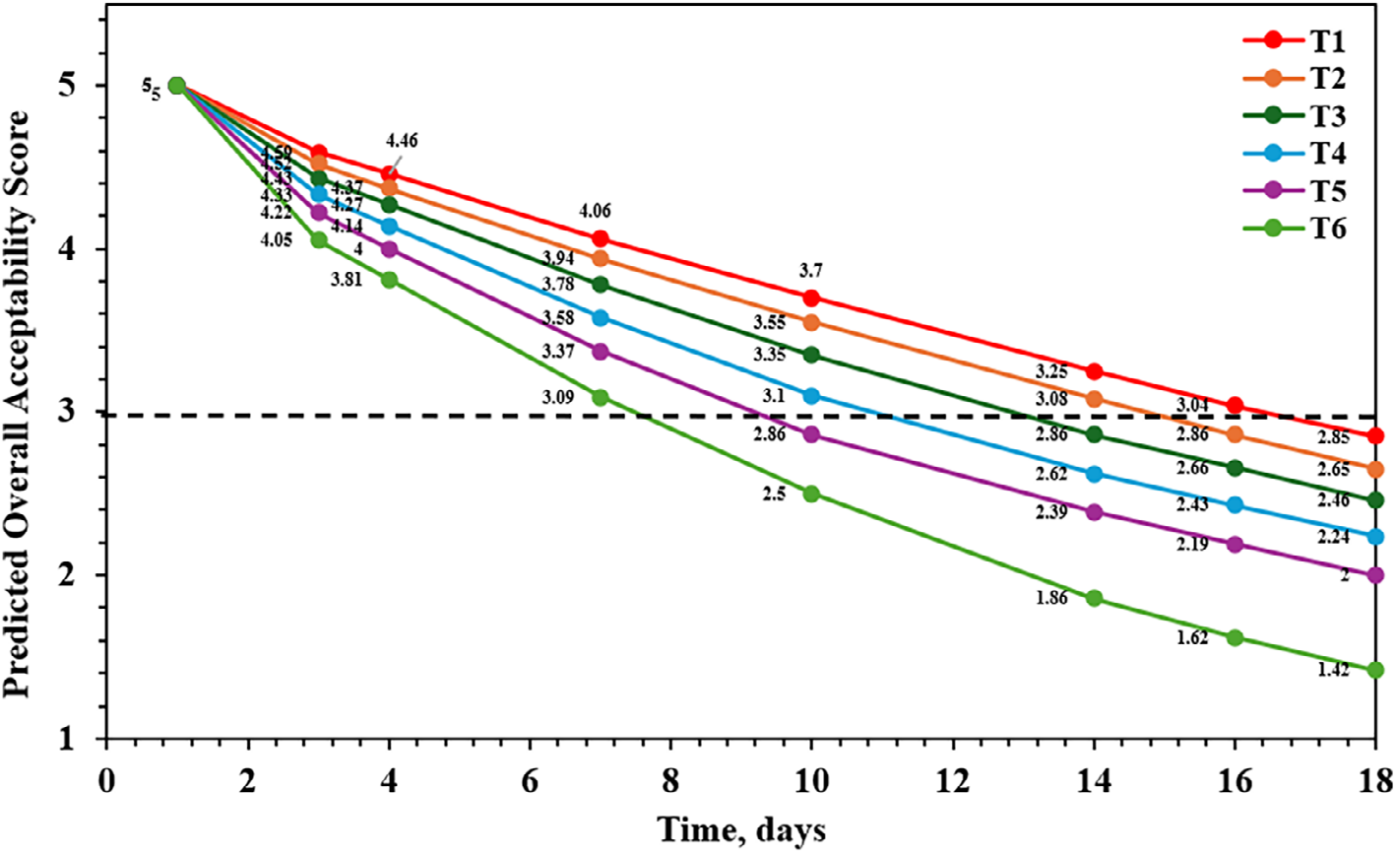
Predicted shelf‐life of six treatments of rocket leaves grown at different levels of nitrogen fertilizer range from 0.005 to 0.015 mol L^−1^ based on overall acceptability at time 0, 3, 4, 7, 10, 14, 16 and 18 days storage at 5 °C. Overall acceptability scale: 5 = uniform dark green leaves with fresh and turgid appearance; 4 = fresh leaves with slight loss of turgidity; 3 = significant loss of turgidity and colour (marketability limit); 2 = significant senescence, wrinkled and yellowish blades; 1 = spoiled with severe wilting, yellowing and decay symptoms. Horizontal dotted line (‐‐‐) shows the limit of market acceptability as 40% loss of the initial appearance quality attributes.

The findings confirm that the secondary model is a strong and biologically significant tool of predicting postharvest sensory degradation as a variable of preharvest nitrogen management. By implementing this model into the context of decision support systems, it may be possible to focus on precision fertilization solutions to maximize the results in terms of product quality and shelf‐life.

### Integrated discussion

This study combined nitrate accumulation, vitamin C degradation, microbial growth, chlorophyll dynamics, texture and sensory quality in order to assess the effect of preharvest nitrogen fertilization on postharvest physiology and shelf‐life of fresh‐cut rocket leaves. A clear dose‐dependent trade‐off developed in which high nitrogen led to improved initial greenness and yield, but poor nutritional quality, safety and storability. Treatments T_5_ and T_6_ (0.013–0.015 mol L^−1^ of nitrogen) produced the highest biomass and chlorophyll but accumulated too much nitrate (11–12 g kg^−1^ FW), which is above EU limits (6–7 g kg^−1^; No. (EU) 2023/915). While the chlorophyll model effectively describes the observed degradation kinetics, its applicability is mainly limited to the defined experimental domain. Direct transferability of these specific model parameters to other crop species, soil‐grown systems, or fluctuating storage temperatures would require local recalibration. Nitrate accumulation was greater in late season harvests, confirming the effect of environmental light and temperature.^24^ High nitrate was associated with an inverse relationship with AA suggesting a redox trade‐off between nitrate assimilation and antioxidant synthesis.^30^, ^31^

Chlorophyll content was increased with nitrogen because of the role nitrogen played in the biosynthesis of chlorophyll, but degradation of chlorophyll during storage was accelerated under high nitrogen. The fitted kinetic model (Eqn 3) from the exponentially decreasing chlorophyll (*C*_0_) and degradation constant (*k*) with increasing nitrogen, as a time exponent *n* = 1.32 indicating non‐linear senescence caused by oxidative stress.^43^ Thus, excess nitrogen produced initially greener leaves, but more unstable so that they lost colour more quickly. Other quality traits also showed the same tendency: high‐nitrogen leaves showed an increased rate of vitamin C loss, microbial growth and texture softening due to very likely increasing tissue succulence and metabolic activity.^45^, ^46^, ^47^ The good correlation between nitrogen concentration and sensory degradation (*k* = 0.0072 + 0.00011*N*; *R* ^2^ = 0.99) indicated that excessive nitrogen almost reduced sensory shelf‐life by half (7.6 days for T_6_ *versus* 17.8 days for T_1_).

Moderate fertilization (T_1_–T_3_; 0.005–0.009 mol L^−1^ of nitrogen) provided the best compromise between regulatory limits for nitrate level, reduced losses of pigments and vitamins, decreased microbial growth and acceptable sensory quality up to 16–18 days. These nitrogen levels probably sustain balanced cell structure, respiration and antioxidant defences.^43^, ^48^ The integrated kinetic approach showed that optimum nitrogen management guarantees metabolic equilibrium and not maximum greenness. Nitrogen concentrations of 0.005 to 0.009 mol L^−1^ are optimal in terms of high visual quality, nutritional value and safety, while higher rates lead to fast degradation and regulation non‐compliance. To contextualize the predictive framework, the preharvest nitrogen concentration in the NS serves as a highly controllable, system‐level input parameter, acting as an integrated proxy for the complex physiological shifts within the leaf tissues. Consequently, the developed models capture the comprehensive, correlative influence of the preharvest nutrient environment on postharvest quality decline, rather than isolating specific localized biochemical mechanisms. This systemic approach provides a highly practical foundation for commercial shelf‐life prediction, while leaving target‐specific tissue metabolic pathways open for future detailed validation. Similarly leaf respiration rates can be affected by preharvest nitrogen fertilization,^18^ therefore potential variations inside package gaseous atmosphere could be a minor yet important co‐founding factor worth monitoring during storage of rocket leaves for further studies. In this study storage in air was designed to avoid any effect of the respiration rate and consequent gas atmosphere inside packaging on the assessed quality parameters. Future improvements to these predictive models should incorporate environmental factors and adaptive nitrogen‐application strategies to maintain both productivity and postharvest quality.

## CONCLUSION

This study showed that preharvest nitrogen fertilization significantly affects the postharvest quality, safety and shelf‐life of fresh‐cut leaves of rocket (*D. tenuifolia*). Integrated kinetic modelling showed that high nitrogen supply (≥ 0.013 mol L^−1^) accelerated chlorophyll and sensory degradation shortening the shelf‐life to approximately 7.6 days compared to 0.005–0.009 mol L^−1^ of nitrogen levels, which maintained sensory acceptability for up to 17.8 days. The developed shelf‐life model (*R* ^2^ > 0.95) offers a practical predictive tool for optimizing nitrogen fertilization and the storage performance.

These findings define an optimal fertilization range that balances yield, nutritional quality, and food safety within regulatory nitrate limits. Beyond technological implications, the results highlight that sustainable nitrogen management can reduce postharvest losses and environmental burdens associated with excessive fertilizer use. Overall, moderating nitrogen inputs represents a dual strategy for enhancing product value and promoting environmental stewardship. Future work should validate these predictive models under commercial packaging and distribution systems.

## AUTHOR CONTRIBUTIONS

Aysha Saleem: data curation (lead); formal analysis (lead); investigation (lead); project administration (lead); writing – original draft (lead). Maria Luisa Amodio: conceptualization (equal); methodology (equal); supervision (lead); funding acquisition (lead); review (equal). Maria Gonnella: writing and editing (equal). Pramod V Mahajan: review and editing (equal); data visualization (equal). Lucia Bonelli: writing and editing (equal). Giancarlo Colelli: conceptualization (equal); methodology (equal); supervision (lead); funding acquisition (lead); review (equal).

## FUNDING INFORMATION

This research was funded by Italian Ministry of University and Research (MUR), project ‘Conservabilità, qualità e sicurezza dei prodotti ortofrutticoli ad alto contenuto di servizio–ARS01_00640–POFACS’, D.D. 1211/2020 and 1104/2021.

## CONFLICT OF INTEREST

The authors declare that they have no known competing financial interests or personal relationships that could have appeared to influence the work reported in this article.

## Data Availability

The data that support the findings of this study are available from the corresponding author upon reasonable request.
